# Supplementary material for: Identification of photoperiod-induced specific miRNAs in the adrenal glands of Sunite sheep (Ovis aries)
Source: Front Vet Sci. 2022 Jul 22;9:888207. doi: 10.3389/fvets.2022.888207 (PMC9354845; doi:10.3389/fvets.2022.888207)
Supplement: Supplementary file 1 [file Data_Sheet_1.ZIP › Supplementary file/miRDeep2 file.zip!/miRDeep2 file/Know_Structure/SP42A1/oar-mir-376a.pdf]

miRBase precursor : oar-mir-376a
Total read count : 72
oar-miR-376a-5p read count : 47
oar-miR-376a-3p read count : 24
remaining reads : 1

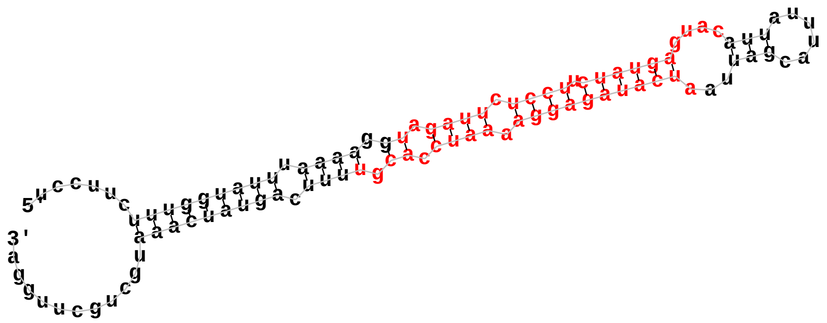

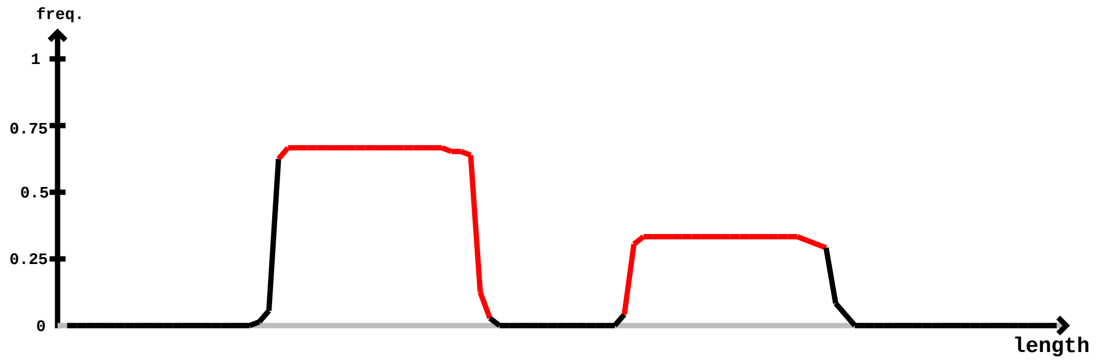


```
                                   oar-miR-376a-5p
                                                                     oar-miR-376a-3p
5'- uccuucuuugguauuuaaaaggguagauucuccuucuaugaguacauuauuuacgauuaaucauagaggaaaauccacguuuucaguaucaaaugcugcuugga  -3'   exp
    ......((((((((.((((.((.((((.((((.((((((....(((......)))...)))))))))).)))).)).)))).)))))))))...........   reads      mm        sample
    ...................agguagauucuccuucuaugagu.............................................................   1          0         seq
    ....................gguagauucuccuucuaugagu.............................................................   2          0         seq
    ....................gguagauucuccuucuaugagua............................................................   1          0         seq
    .....................guagauucuccuucuaug................................................................   1          0         seq
    .....................guagauucuccuucuaugag..............................................................   1          0         seq
    .....................guagauucuccuucuaugagu.............................................................   32         0         seq
    .....................guagauucuccuucuaugagua............................................................   5          0         seq
    .....................guagauucuccuucuaugaguac...........................................................   2          0         seq
    ......................uagauucuccuucuaugagu.............................................................   2          0         seq
    ......................uagauucuccuucuaugagua............................................................   1          0         seq
    .........................................................aaucauagaggaaaauccacg.........................   1          0         seq
    .........................................................aaucauagaggaaaauccacgu........................   2          0         seq
    ..........................................................aucauagaggaaaaucca...........................   1          0         seq
    ..........................................................aucauagaggaaaauccac..........................   1          0         seq
    ..........................................................aucauagaggaaaauccacgu........................   13         0         seq
    ..........................................................aucauagaggaaaauccacguu.......................   3          0         seq
    ..........................................................aucauagaggaaaauccacguuu......................   1          0         seq
    ...........................................................ucauagaggaaaauccacguuu......................   2          0         seq
```
